# P24 Point of care rapid urinary tract infection phenotypic antibiotic susceptibility test

**DOI:** 10.1093/jacamr/dlad077.028

**Published:** 2023-08-02

**Authors:** Megan Roegner, Megan Butler, Paula Millirons

**Affiliations:** GeneCapture Inc., USA; GeneCapture Inc., USA; GeneCapture Inc., USA

## Abstract

**Background:**

Urinary tract infections (UTIs) are one of the most common infections in the world today. In the last few years, the occurrence of antimicrobial resistant pathogens causing UTIs has rapidly increased. One in three UTIs are trimethoprim/sulfamethoxazole (Bactrim) resistant, and one in five are resistant to other common antibiotic choices. This growing resistance is mainly due to overuse or misuse of antibiotics. The strongest tool the medical community has available to combat antimicrobial resistance (AMR) is rapid, correct application of antibiotics to an identified bacterial infection. Clinics and physician offices seek on-site rapid solutions to speed the pathway to timely and appropriate antibiotic therapies. GeneCapture is developing products that perform rapid UTI pathogen identification and antimicrobial susceptibility tests (ASTs) directly from infected urine samples. The AST provides a susceptible versus non-susceptible call in under 2 h and can be applied to samples with single or mixed pathogens. A portable tabletop instrument and disposable cartridge are being engineered to run this test at the point of care, allowing same-day prescriptions. The process is currently operational on the lab bench.

**Methods:**

Fluorescent labelling of bacterial species, followed by optical counting, determines population growth with/without a suite of antibiotics. An on-board algorithm instantly converts raw cell numbers into a differential call of susceptible or non-susceptible for each bug/drug pair. A disposable cartridge with on-board antibiotics has been designed and is being finalized for fabrication. The optical counting is operating in a desktop device.

**Results:**

Testing to date includes 124 strains from 7 of the most common UTI and wound pathogens against a panel of six antibiotics. A total of 541 antibiotic/bacterial strain pairs in single or multiple pathogen antibiotic susceptibility testing produce an initial 88.7% agreement to gold standard culture results. These results are all generated in 2 h or less of assay time and represent a strong preliminary dataset.

**Conclusions:**

This 2 h portable, novel, phenotypic AST profiling will produce informed and effective treatment options for medics, clinics and physician offices long before existing AST methods could return results.

